# The Bitter Fate of the Sweet Heart: Impairment of Iron Homeostasis in Diabetic Heart Leads to Failure in Myocardial Protection by Preconditioning

**DOI:** 10.1371/journal.pone.0062948

**Published:** 2013-05-15

**Authors:** Vladimir Vinokur, Eduard Berenshtein, Baruch Bulvik, Leonid Grinberg, Ron Eliashar, Mordechai Chevion

**Affiliations:** 1 Department of Biochemistry and Molecular Biology, Institute of Medical Research Israel-Canada (IMRIC), The Hebrew University of Jerusalem, Jerusalem, Israel; 2 Department of Otolaryngology, Hadassah-University Hospital, Jerusalem, Israel; Queen Mary University of London, United Kingdom

## Abstract

Cardiovascular dysfunction is a major complication of diabetes. Examining mechanistic aspects underlying the incapacity of the diabetic heart to respond to ischemic preconditioning (IPC), we could show that the alterations in iron homeostasis can explain this phenomenon. Correlating the hemodynamic parameters with levels of ferritin, the main iron storage and detoxifying protein, without and with inhibitors of protein degradation, substantiated this explanation. Diabetic hearts were less sensitive to ischemia-reperfusion stress, as indicated by functional parameters and histology. Mechanistically, since ferritin has been shown to provide cellular protection against insults, including ischemia-reperfusion stress and as the basal ferritin level in diabetic heart was 2-fold higher than in controls, these are in accord with the greater resistance of the diabetic heart to ischemia-reperfusion. Additionally, during ischemia-reperfusion, preceded by IPC, a rapid and extensive loss in ferritin levels, during the prolonged ischemia, in diabetic heart but not in non-diabetic controls, provide additional substantiation to the explanation for loss of respond to IPC. Current research is shedding light on the mechanism behind ferritin degradation as well, suggesting a novel explanation for diabetes-induced loss of cardioprotection.

## Introduction

Despite recent advancements in the prevention and treatment of heart disease, cardiac ailments continue to be a leading cause of mortality in the Western world [1]. Amongst various techniques studied to protect the heart against the consequences of coronary stenosis and occlusion, IPC is considered to have therapeutic potential for reducing myocardial injury during cardiac surgery, percutaneous transluminal coronary angioplasty (PTCA) and heart transplantation [2], [3].

Some publications have documented worse recovery of heart function following prolonged ischemia, in diabetic patients, as in diabetic animals, compared to non-diabetic hearts. Other published lines of evidence have clearly demonstrated the opposite - a significantly better recovery of the diabetic than the non-diabetic heart, after prolonged ischemia. Indeed, the literature is not consistent with studies showing enhanced tolerance of diabetic hearts to prolonged ischemia and reperfusion-injury [4], [5], while others contradict this finding [6]. The limited cardio-protective resources of the diabetic heart were discussed in a recent review [7]. Paradoxically, the diabetic heart demonstrates a poor capacity to respond to IPC [8], [9], [10], in contrast to the non-diabetic heart, where IPC is highly protective.

Various mechanisms have been demonstrated to explain the functional protection provided by pre-conditioning procedures, including ischemic pre-conditioning (IPC). Earlier, we showed that ischemia caused mobilization and redistribution of iron in the myocardium, in an ischemia-duration-dependant manner. This tended to aggravate the ‘reperfusion injury’ [11], [12]. Preventing the redox-cycling activity of the mobilized labile iron, thus conferring protection against reperfusion injury, was attained by the ‘packaging’ of labile iron within ferritin - the major iron storage and detoxifying protein. Our recent findings on IPC showed that IPC led to the accumulation of cellular ferritin and activation of multi-step iron-based mechanism of myocardial protection against reperfusion injury [13].

In the present study, we address the following: (i) Is the diabetic heart as sensitive to ischemia as a non-diabetic myocardium? (ii) Whether the proposed iron-based mechanism of IPC protection is valid also in the diabetic heart and, (iii) Whether the diabetes-induced alterations in this mechanism can explain the poor response of the diabetic heart to IPC.

## Materials and Methods

### Animals

All the experimental protocols have been approved by the Institutional Animal Care and Use Committee of the Hebrew University of Jerusalem, conforming to the Guide for the Care and Use of Laboratory Animals published by the US National Institutes of Health (NIH Publication No. 85–23, revised 1996).

Sprague-Dawley male rats (250–275 g) housed under standard conditions (12 hlight/12 h dark) and fed regular *ad libitum* normal diet and water were used.

### Diabetes Mellitus Model

Rats were made diabetic by a single i.p. injection of streptozotocin (STZ) (Zanosar®, Pharmacia and Upjohn Company, Kalamazoo, MI, USA) (70 mg/kg body weight, in saline). The control group was injected i.p. with equal volume of saline. Typically, a week after STZ injection, glucose level exceeded 300 mg/dl and the animals were considered diabetic. In order to have diabetic-associated systemic effects and to allow for diabetic complications to develop, the heart perfusion experiments were conducted 4 weeks after STZ injection.

### Heart Perfusion and Monitoring of the Hemodynamic Parameters

On the day of the experiment, rats were injected with sodium heparin (500 units/kg i.p.) and after 30 min –80 mg/kg ketamine and 5 mg/kg xylazine mixture i.p. Upon bilateral thoracotomy, hearts with a segment of the ascending aorta were rapidly excised, put in ice-cold heparinized saline, then mounted onto the Langendorff apparatus and perfused using a modified Krebs-Henseleit (KH) buffer [13], [14]. Heart rate (HR), end diastolic pressure (EDP), developed pressure (DP), and its derivatives (+dp/dt and −dp/dt) were recorded. All primary data were processed using a customized version of LabView 7.1 software (National Instruments, Austin, TX, USA). Work index (WI = Heart Rate×DP) was used as an indicator of heart contractility. The degree of cardioprotection was expressed by the percent (ratio) of two values: WI at the completion of the experiment (at the 120^th^ min) and WI at the completion of the stabilization phase (at the 10^th^ min).

### Experimental Protocols

**Figure 1:**
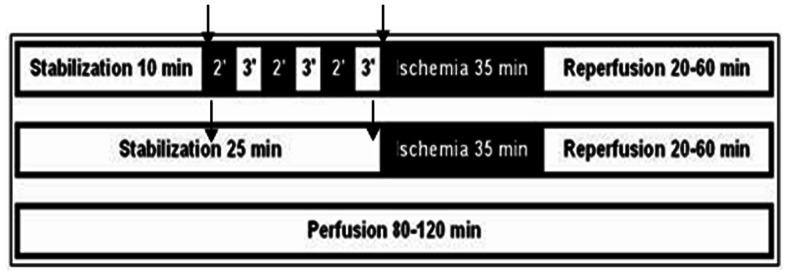
The 3 basic experimental protocols. (i) continuous perfusion (lower bar), (ii) ischemia/reperfusion (I/R) (middle bar) and (iii) ischemic pre-conditioning (IPC) followed by I/R (upper bar).

Three basic experimental protocols were employed (Figure 1) – (i) continuous (un-interrupted) perfusion (PER), (ii) stabilization (25 min) followed by ischemia and reperfusion (35 and 60 min respectively) (I/R), and (iii) stabilization (10 min) followed by IPC, ischemia and reperfusion (IPC+I/R). The hemodynamic parameters of the heart were monitored and recorded continuously throughout, during all experimental protocols. Under the I/R protocol, the stabilization phase was extended from 10 to 25 min to compensate for the 15 min of the IPC procedure, which consisted of 3 cycles of (2 min ischemia and 3 min perfusion). Biochemical indices, such as heart tissue levels of ferritin and its saturation with iron, were measured using heart tissue samples at four time points: (i) end of stabilization (10 or 25 min), (ii) completion of the IPC procedure (25 min), (iii) end of ischemia (60 min), and (iv) end of reperfusion (120 min) [13]. At each time-point 7–13 hearts were used and analyzed to meet the criteria of adequate statistical analysis.

In order to test the roles of ferritin during the post-IPC prolonged ischemia, group diabetic animals’ hearts was treated with inhibitors of proteasomal and/or lysosomal pathways of protein degradation., This was accomplished by 3 min perfusion with KH buffer containing MG-132 (6 µM) (proteasomal inhibitor) and/or, leupeptin (10 mg/liter) and pepstatin (6 mg/liter) (lysosomal proteases inhibitors) (all the chemicals were purchased from Sigma, St. Louis, MO, USA), at the end of stabilization or at the end of IPC (Figure 1). Ferritin was measured in heart tissue samples, at the same 4 pre-determined time points as described above.

#### Infarct size

Infarct size (IS) was measured at the completion of each of the protocols [15], [16], [17], using staining with Evans Blue (EB) and triphenyltetrazolium chloride (TTC) (Sigma, St. Louis, MO, USA). The area at risk (EB negative) and the size of the infarcted tissue (TTC negative) were digitally photographed with a Nikon Coolpix 5000 camera and quantified with IMAGE J 1.32 (NIH, USA) software.

#### Heart homogenates

Heart homogenates were prepared using a Cole Parmer Teflon homogenizer (Chicago, IL). About 100 mg wet tissue was homogenized in 1.6 ml of a lysis buffer containing Tris-HCl 50 mM, cysteine 1 mM, sodium citrate 1 mM and MnCl_2_ 0.5 mM, phenyl-methyl-sulfonyl-fluoride (PMSF) 0.25 mM, digitonin 0.02%, at pH7.6. Protein concentrations in the homogenate were determined using a BCA (BiCinchoninic Acid) kit according to the manufacturer’s (Pierce, USA) instructions. The same ferritin levels were found in both left and right parts of the heart (Berenshtein E & Chevion M, unpublished results).

#### Ferritin (Ft) levels

Ferritin (Ft) levels in the homogenates were determined using an ELISA-based “sandwich” assay developed in this laboratory [13]. Briefly, antibodies against L and H rat ferritin were prepared as described earlier [18]. ELISA 96-well micro plates, pre-coated with goat anti-rat L-Ft were blocked with 0.5% gelatin and 0.1% sodium azide followed by sample application. Rabbit anti-rat H-Ft was used as the secondary antibody. Plates were treated with goat anti-rabbit IgG conjugated to β-galactosidase. Chlorophenol Red-β-D-Galactopyranoside was then added at a concentration of 0.35 mg/ml and the plates scanned using a microplate reader (Dynatech MR 5000, Boonville, IN, USA) with a test (570 nm) and reference (630 nm) filters.

### Ferritin-bound Iron

Ferritin was immuno-precipitated using a mixture of anti-H and anti-L ferritin antibodies. The precipitate was dissolved in nitric acid and its iron content determined by either Zeeman atomic absorption spectrometer [13] or spectrophoto-metrically with batho-phenanthroline bi-sulphonate (BPS) [19].

### qRT-PCR Measurements

qRT-PCR measurements were performed according to previously published protocols [20]. Primers for the target genes and the housekeeping gene β-Actin were designed using Primer3 software (from: http://frodo.wi.mit.edu/cgi-bin/primer3/primer3_www.cgi). All genes products were normalized according to the level of β-Actin. Changes in gene expression were calculated using the 2^(–ΔΔCT)^ method [21].

### Statistical Analysis

The data were analyzed using repeated one-way ANOVA followed by the Scheffe post hoc test, for multiple comparisons (with α = 0.05). Statistically significant differences were considered when p≤0.05.

## Results

Rats were rendered diabetic, with i.p. injection of streptozotocin (STZ). Their blood glucose levels and body weights were monitored for a four-week period, prior to the *ex vivo* heart experiments, and are given in Table S1 of the supporting data.

### Diabetic Hearts are more Resistant to I/R - Hemodynamics

Isolated diabetic hearts (DH) were significantly more resistant to prolonged ischemia and reperfusion (I/R protocol described in Figure 1) than their matched controls, as indicated by the hemodynamic parameters (Table 1). While control hearts subjected to I/R had lost 65% of their basal WI capacity, by the completion of the reperfusion phase, the diabetic hearts lost only 44%. Analogously, the developed pressure (DP) recovered to (53±5)% of the pre-ischemic value in DH, and only to (34±6)%, in the control group (p<0.05) (Table 1). The end diastolic pressure values (EDP) after 120 min of un-interrupted Perfusion were similar in both diabetic and control groups. Likewise, at the end of the I/R protocols, the EDP values were the same. Consistently with the literature [22], DH showed clear bradycardia; (HR_0_ averaging at 220 min^−1^) were considerably slower than the controls (averaging at 270 min^−1^). DH showed no gain of function following IPC; their WI recovery for IPC+I/R group were similar to (but smaller than) those for I/R group. In contrast, the hemodynamic parameters of the IPC+I/R control hearts were markedly better than after I/R (without IPC).

**Table 1:** The hemodynamic parameters of rat hearts perfused *ex vivo* according to the three basic protocols – un-interrupted Perfusion, I/R and IPC+I/R without and with cocktail of inhibitors of proteins degradation.

Group	Protocol	N	DP_0_ mm Hg	HR_0_ min^−1^	DP %	WI %	EDP_120_ mm Hg
Control	Perfusion	7	114±12	268±17	84±4	83±4	0.4±0.1
Control	I/R	9	111±7	260±6	34±6	35±9	51±10
Control	IPC+I/R	7	97±5	283±7	87±4*	88±8*	8±2
Diabetes	Perfusion	9	103±9	214±9‡	78±8	88±9	1.6±0.6
Diabetes	I/R	13	103±7	218±7‡	53±5#	56±8#	47±6
Diabetes	IPC+I/R	12	110±8	227±8‡	58±5	52±4	36±5†
Diabetes+IPD	I/R	5	115±8	203±25	23±1	22±3	16±15
Diabetes+IPD	IPC+I/R	5	119±3	200±17	71±7*	72±8*	23±15

The hemodynamic recovery of the work index (WI) and the developed pressure (DP) at the completion of each of the protocols, was compared to the pre-ischemic value. The following additional abbreviation are used: EDP_120_– end diastolic pressure at the completion of the experiment - 120 min; WI – work index; N - the number of hearts in the group; DP_0_ and HR_0_– developed pressure and heart rate at the stabilization phase; IPD – cocktail of inhibitors of protein degradation.

Mean±SEM are shown.

* p<0.01 *vs.* I/R data in control;

# p<0.05 *vs.* I/R data in control;

† p<0.05 *vs.* IPC+I/R data in control;

‡ p<0.01 *vs.* the respective groups in control.

* p<0.005 *vs.* I/R;

# p<0.05 *vs.* I/R.

To examine whether a direct interaction between STZ and the heart occurred and may affect myocardial function, hearts of animals that had developed moderate diabetes by STZ, (blood glucose levels of 180–250 mg/dl) were monitored. No significant differences in the basal hemodynamic heart characteristics were observed between the moderately diabetic animals and the control group. WI recovery at the completion of IPC+I/R protocol was similar to the recovery of the controls, (78±7)% *versus (*88±8)%, respectively, and (46±7)% *versus* (35±9)% without prior IPC.

### Diabetic Hearts are more Resistant to I/R - Histology

Analogous results have been demonstrated using histology data: for I/R protocols, the area at risk (AAR) of DH were measured at the completion of the experiments (after reperfusion), and were found significantly smaller than those for the controls, (49±6)% and (75±4)%, respectively. Since prolonged global ischemia was used in these experiments – most investigators consider that the entire heart is at risk, and AAR = 100%.

Infarct size values (IS) were calculated relative to the total volume of the heart (considering AAR = 100%), and relative to the AAR which was actually measured at the end of the experimental protocol (after reperfusion) by (Tetrazolium chloride (TTC) staining. For the I/R protocols, IS values were similar for both the control and DH groups ((19±6)% and (13±1)% and (25±5)% and (27±2)%), respectively (Table 2)). Unlike these results, for the IPC+I/R protocols, a significantly smaller AAR value was obtained for the control hearts, than for DH (27±1)% and (72±11)%, respectively. Consistently, in the DH subjected to IPC+I/R, IS calculated by both methods indicated a higher degree of dysfunction than their non-diabetic control counterparts.

**Table 2:** The histology parameters of rat hearts perfused *ex vivo* according to the three basic protocols – un-interrupted Perfusion, I/R and IPC+I/R.

Control Hearts	I/R	IPC+I/R
AAR (%)	75±4	27±1
IS, calculated as a % of AAR	25±5	15±2
IS, calculated as a % of a total heart volume	19±6	4±1
Diabetic Hearts		
AAR (%)	49±6*	72±11*
IS, calculated as a % of AAR	27±2	38±4*
IS, calculated as a % of a total heart volume	13±1	27±3*

AAR - the area-at-risk is expressed as percentage of the total slice area; IS - the infarct size. Mean±SEM are shown.

* p<0.05 *vs.* IPC+I/R data in control.

### Ferritin in the Ischemic Hearts

**Figure 2:**
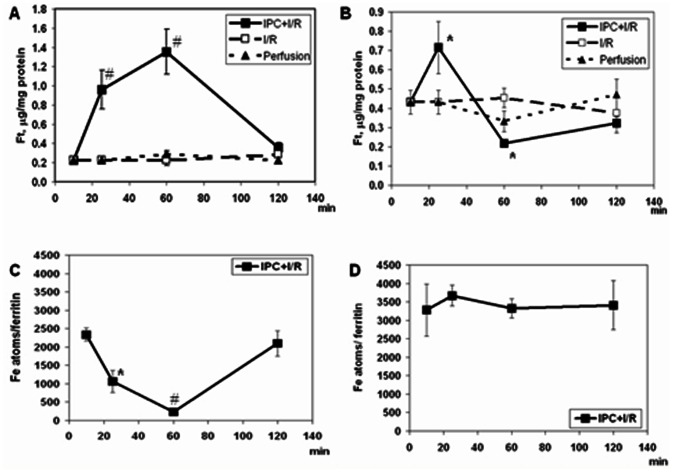
Ferritin levels (Panels A and B) and ferritin saturation with iron (Panels C and D) in hearts of non-diabetic and STZ-diabetic rats. Under I/R protocol (□), the hearts were stabilized for 25 min followed by global ischemia (35 min) and reperfusion (60 min). Under IPC+I/R protocol (•), the stabilization phase (10 min) was followed by the IPC procedure (15 min of intermittent ischemia (2 min) followed by perfusion (3 min)), prolonged ischemia and reperfusion. The perfusion protocol (▴) was run to detect spontaneous changes in Ft during the entire protocol of 120 min. Mean±SEM are shown. ^#^ denotes p<0.001 *versus* the values for either I/R or Perfusion; * denotes p<0.01 *versu*s either I/R or Perfusion; **^** denotes p<0.05 *versus* either I/R or Perfusion.

Cellular ferritin content and the average number of iron ions within a single ferritin molecule (N_Fe_) were measured (Figure 2). Subjecting non-diabetic hearts to IPC resulted in a marked increase in heart ferritin levels during the IPC procedure, which remained high throughout the subsequent prolonged ischemia phase (Figure 2; panel A and references [13], [23]), and returned to the baseline value during the reperfusion. It is important to note that in DH, the baseline level of ferritin was twice that of the controls ((0.39±0.09) µg/mg in DH as compared to (0.20±0.08)µg/mg (p = 0.013), in the controls). Furthermore, the IPC procedure in DH yielded an additional two-fold increase in ferritin. Unlike in the controls, however, during the subsequent prolonged ischemia, DH ferritin level dropped by more than four-fold, reaching a value below 0.20 µg/mg (panel B).

When hearts, DH or controls, were subjected to I/R, their ferritin level showed no change along the entire duration of the experiment. Indeed, the level of ferritin in DH remained twice as high as in control hearts ((0.41±0.05) *versus* (0.20±0.05) µg/mg protein, p<0.02)). This higher ferritin level in the diabetic myocardium could explain the higher resistance of the DH to I/R, when compared to I/R in control hearts. Since the extra ferritin level, gained by DH during the IPC procedure, was lost during the subsequent prolonged ischemia (Figure 2B), at the onset of reperfusion, the moment of highest risk, ferritin levels in the DH were lower than its baseline level, explaining the poor recovery of DH following IPC+I/R.

### Number of Iron Atoms Bound to Ferritin

N_Fe_ is the average number of iron atoms bound to a single ferritin molecule In controls (Figure 2C), the changes in N_Fe_ levels along the IPC+I/R protocol mirrored those of the changes in ferritin levels (Figure 2A and references [13], [23]). In the diabetic hearts (panel 2D) no changes in N_Fe_ were detected along the entire IPC+I/R protocol.

### Ferritin mRNA in Diabetic and Non-diabetic Heart

The mRNA levels of ferritin subunits (H-Ft and L-Ft) were measured by qRT-PCR (Table 3). In DH, unlike in the controls, IPC caused a marked decrease in H-Ft mRNA – the message for the dominant ferritin subunit in the heart, while no change was observed in L-Ft mRNA. In control hearts the levels of L-Ft mRNA increased markedly to 3.76-fold of its basal level.

**Table 3:** mRNAs levels of H- and L-subunits of ferritin in rat hearts subjected ex vivo to IPC+I/R protocol.

		Protocol step			
Parameter	Group	Stabilization	IPC	IPC+Ischemia	IPC+I/R
H-Ft mRNA (a.u.)	Control	1.00±0.07	1.01±0.10	1.17±0.15	0.82±0.32
H-Ft mRNA (a.u.)	Diabetes	1.35±0.20	0.59±0.11*	1.33±0.20	1.40±0.65
L-Ft mRNA (a.u.)	Control	1.00±0.16	1.35±0.29	3.76±0.69^∧^	1.47±0.18
L-Ft mRNA (a.u.)	Diabetes	1.57±0.22	1.49±0.16	1.30±0.20*	1.86±0.29

After 10 min stabilization hearts were subjected to IPC (15 min) followed by prolonged global ischemia (35 min) followed by reperfusion (60 min). mRNA relative levels in the control group were set to 1.00, for either H-Ft and L-Ft.

Mean±SEM are shown.

* - denotes p<0.05 *versus* the control;

^ - denotes p<0.05 *versus* the stabilization phase of the controls.

### Effect of Protease Inhibitors

**Figure 3:**
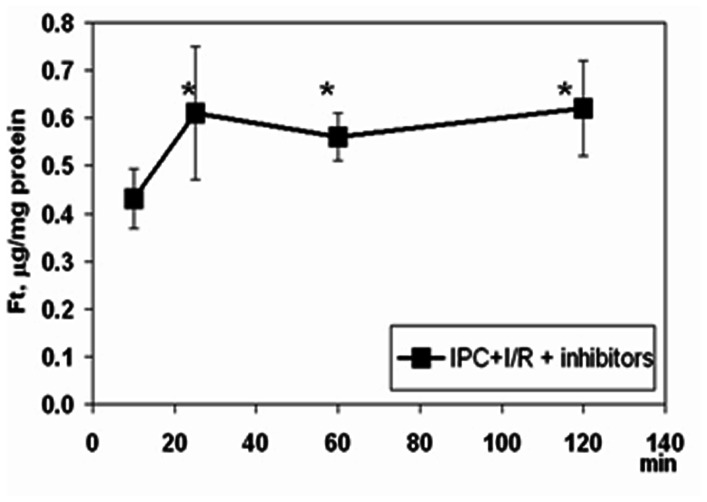
Ferritin (Ft) levels in hearts from STZ-induced diabetic rats subjected to ischemic preconditioning followed by prolonged ischemia and reperfusion (IPC+I/R protocol) when treated with a cocktail of proteases inhibitors. Mean±SEM are shown. * - denotes p<0.01 *versus* the mean value at 10 min. # - denotes p<0.05 *versus* the treated group at the same time point along the protocol.

In order to halt the degradation of the high level of IPC-induced ferritin, along the experiments, the inhibitors of protein degradation were introduced to the heart via the perfusate (Figure 1). The cocktail, containing proteasomal and lysosomal inhibitors, led to the restoration of the protective effect of IPC in the diabetic animals (Table 1). When the cocktail was introduced to the beating heart, before the IPC procedure, ferritin (protein) level remained high until the end of the protocol (Figure 3). This was in accord with our working hypothesis that high ferritin level during ischemia and the onset of reperfusion well correlates with myocardial protection.

The effect of the cocktail on mRNA levels of ferritin-subunits was investigated. Under the same conditions, the pattern of L-Ft mRNA levels, in DH, resembled the pattern observed in the control hearts: an increase during IPC, which persisted during the subsequent prolonged ischemia, but became very low during the reperfusion phase reaching the initial (stabilization) level of the control group (Table 4).

**Table 4:** mRNAs of ferritin subunits in diabetic rat hearts subjected to IPC+I/R protocol and treated with a cocktail of proteases-inhibitors (after the IPC procedure).

	Perfusion 25′	IPC	IPC+I	IPC+I/R
**H-ferritin mRNA**	1.2±0.2	0.8±0.2*	0.97±0.2*	1.0±0.1
**L-ferritin mRNA**	1.0±0.2	2.67±0.5*	4.5±1.0*	1.4±0.5

After 10 min stabilization hearts were subjected to IPC (15 min) followed by perfusion (3 min) with KH containing the cocktail of protease inhibitors, and then subjected to global ischemia (35 min) and reperfusion (60 min) (see Material and Methods). Ferritin mRNA levels for the control group were set to 1.00, for both H- and L-subunits. Mean ± SEM are shown;

* - denotes p<0.05 *versus* the respective value for 25 min Perfusion.

Furthermore, introducing the cocktail of inhibitors, was associated with a partial restoration of the IPC protection, as indicated by the post-ischemic functional recovery, with WI reaching (72±8)%, which represented a significant improvement over the non-treated DH (with WI recovery of (52±4)%) (Tables 1 and 5). Interestingly, when DH were subjected to I/R (without prior IPC) - the cocktail of inhibitors interfered with enhanced post-ischemia recovery (WI remaining at only (22±3)%) (Tables 1 and 5).

**Table 5:** The hemodynamic parameters of diabetic rat hearts perfused *ex vivo* according to the I/R and IPC+I/R protocol, in absence and presence of the inhibitors of proteins degradation.

Group	With no inhibitor	Cocktail of inhibitors 10′	Proteasomal inhibitor at 10′ (before IPC)	Lysosomal inhibitorat 10′ (before IPC)	Proteasomal inhibitor at 25′ (after IPC)	Lysosomal inhibitor at 25′ (after IPC)
I/R	56±8	22±3*	62±14	79±10*	75±7*	76±12*
IPC+I/R	52±4	72±8*	101±17	52±11	33±9*	39±1*

The hemodynamic recovery of the work index (WI), at the completion of each protocol was compared to the analogous value without inhibitors.

Mean ± SEM are shown.

* p<0.05 *versus* the values, obtained without the inhibitors of proteins degradation.

Adding a proteasomal inhibitor (MG-132) to the perfusate, immediately *before* the IPC procedure, led to complete post-ischemic functional recovery in DH (Table 5), while the recovery of DH subjected to IPC+I/R, without the inhibitor, remained compromised (WI = (52±4)%).

In contrast, adding MG-132 *after* the IPC procedure caused marked suppression of the post ischemic recovery of the DH subjected to proteasomal inhibition+IPC+I/R, with WI = (33±9)%. When the lysosomal inhibitors were added *before* the IPC procedure – no effect on the hemodynamics of the DH was noticed. If the lysosomal inhibitors were added *after* the IPC procedure, the WI reached at the completion of the experiment was moderately lower. In control experiments, without IPC, inhibitors of either pathway (proteasomal or lysosomal) were introduced. Neither of them had any effect on heart function (data not shown).

## Discussion

Diabetes accelerates the atherosclerotic process, frequently by reducing blood flow to the cardiac muscle [24], [25], [26]. Repeated episodes of myocardial ischemia and reperfusion may lead to preconditioning of the heart, allowing the myocardium to better withstand subsequent severe ischemia and to avoid, or at least reduce, the consequences of a myocardial infarction. A number of human and animal studies indicated that the resistance of diabetic hearts to prolonged ischemia might be higher than that of non-diabetics [27], [28], [29]. This is in accord with the proposal that the diabetic heart is under continuous partial “chronic preconditioning” [30].

Unexpectedly, we found that subjecting the diabetic heart to IPC not only did not confer protection, but also rendered the diabetic heart more sensitive to prolonged ischemia and reperfusion than without prior IPC.

The present study was focused on functional and mechanistic aspects of the IPC response of the diabetic myocardium. Earlier, we proposed a mechanism of IPC protection of the heart involving mobilization of labile iron and neutralization of its redox activity by ferritin [13]. Here we showed that the ‘resting’ diabetic myocardium contained double the amount of ferritin as compared to the non-diabetic control heart. When subjected to I/R, this elevated ferritin level, which persisted over the period of prolonged ischemia and reperfusion, correlated with the better post-ischemic recovery of the diabetic versus non-diabetic heart. Unlike in the control heart, when the diabetic heart was subjected to IPC+I/R, the elevated level of ferritin dropped four-fold during the prolonged ischemia and was very low at the moment of highest risk- the onset of reperfusion. The loss of the capacity of the diabetic heart to respond to IPC stems, to a large extent, from the myocardial inability to maintain the high level of ferritin during the ischemia phase. This is in accord with published data demonstrating enhanced protein turnover in diabetes in general, and, in particular, under ischemia [31], [32], [33], The correlation between the inhibition of ferritin degradation (Figure 3) and the restoration of IPC-protection under perfusion, with inhibitors of protein degradation, (Table 5) corroborates this idea.

Ferritin synthesis is regulated at both transcriptional and post-transcriptional levels. Under *in vivo* oxidative stress and in the presence of labile iron, a long-lasting increase in gene transcription of both H- and L-subunits of ferritin is observed [34]. In the present study, a marked increase in L-Ft gene expression was detected in control hearts at the completion of IPC and prolonged ischemia, without reperfusion (after IPC+I), indicating transcriptional control of ferritin (Table 3). The *de novo* ferritin, synthesized during the prolonged ischemia phase, was L-subunit-rich, even though heart ferritin, in general, is an H-rich protein [35], [36]. In the diabetic hearts, the increase in ferritin during IPC was associated with a concomitant *decrease* in the amount of H-subunit mRNA. This L-subunit-abnormally enriched ferritin could conceivably be amenable to a more rapid degradation during the reperfusion phase (Figure 2B). An analogous finding was observed by others: after 25 min ischemia, total mRNA was reduced ∼2-fold while the levels of most of myocardial proteins examined increased [37].

Elevated levels of ferritin protect against oxidative stress, including reperfusion injury [13], [23]. Indeed, support of this general view may be found in various systems [38], [39], [40]. The data concerning the use of inhibitors of protein degradation further supported this concept; the cardio-protective effect of IPC in DH was nearly completely maintained, concomitantly with the capacity of ferritin to scavenge labile iron, thus preventing its diabetes-induced degradation.

The results strongly support the likelihood that labile iron and ferritin play a significant role in the response of control and diabetic hearts to IPC. Excessive ferritin in the diabetic heart successfully stored and detoxified the diabetes-associated iron redundancy, conferring a ‘chronic (partial) preconditioning’ effect, enhancing the tolerance toward ischemia in un-preconditioned diabetic hearts. The failure of the diabetic heart to maintain excess ferritin concentration, which is required for IPC cardio-protection, explains the incapacity to respond to IPC [31], [41]. On the other hand, perfusion of un-preconditioned diabetic hearts with a cocktail of inhibitors of protein degradation halted the removal of oxidized and glycated proteins from the diabetic hearts (Tables 1 and 5). These observations pave the way for further studies on ‘chronic preconditioning’ and acute ischemia in non-diabetic, non-iron-overloaded hearts, in which baseline levels of ferritin are increased through administration of NO-donors [42], [43] or HIF-2α inhibitors [44], mimicking the ‘beneficial’ yet unusual influence of the diabetic state.

## Supporting Information

Table S1 **Body weights and blood glucose levels in STZ-injected rats and their matched controls along the 4-week experiments.**
(DOC)
