# Supplementary material for: The Bitter Fate of the Sweet Heart: Impairment of Iron Homeostasis in Diabetic Heart Leads to Failure in Myocardial Protection by Preconditioning
Source: PLoS One. 2013 May 15;8(5):e62948. doi: 10.1371/journal.pone.0062948 (PMC3655153; doi:10.1371/journal.pone.0062948)
Supplement: Table S1 — Body weights and blood glucose levels in STZ-injected rats and their matched controls along the 4-week experiments. (DOC) [file pone.0062948.s001.doc]

**Supplemental materials**

**Table S1:**  **Body weights and blood glucose levels in STZ-injected rats and their matched controls along the 4-week experiments**

| **Parameter** | **Group** | **Days** | | | |
| --- | --- | --- | --- | --- | --- |
|  |  | **0** | **14** | **21** | **28** |
| **Body weight, g** | **Control** | **245±8** | **266±6** | **272±6** | **279±8** |
|  | **Diabetes** | **253±8** | **245±6*** | **250±7*** | **240±10**** |
| **Blood glucose mg/dl** | **Control** | **79±9** | **77±9** | **80±8** | **78±8** |
|  | **Diabetes** | **81±10** | **358±44^** | **376±45^** | **438±56^** |

Figure Legend for Supplementary Table S1

Rats were injected with STZ (day1) and became severely diabetic, as evinced by their blood glucose levels (BGL), by days 14 - 28. This was accompanied by retardation of weight gain along the experiment, when compared to non-diabetic controls. By the end of week 4 the animals were used for heart perfusion experiments, *ex vivo*.

Values represent Mean±SEM. ***** - denotes p < 0.05, ****** denotes p < 0.01 and **^** denotes p < 0.0001 for the difference between the two groups.
